# Intact vestibular function is relevant for anxiety related to vertigo

**DOI:** 10.1007/s00415-019-09351-8

**Published:** 2019-05-09

**Authors:** J. Decker, K. Limburg, P. Henningsen, C. Lahmann, T. Brandt, Marianne Dieterich

**Affiliations:** 1 Department of Neurology, Klinikum Grosshadern, University Hospital, Ludwig-Maximilians-Universität München, Marchioninistrasse 15, 81377 Munich, Germany; 2 German Center for Vertigo and Balance Disorders, University Hospital, Ludwig-Maximilians-Universität München, Munich, Germany; 3 Department of Psychology, Ludwig-Maximilians-Universität München, Munich, Germany; 4 Department of Psychosomatic Medicine and Psychotherapy, Klinikum Rechts Der Isar, Technische Universität München, Munich, Germany; 5 Department of Psychosomatic Medicine, University of Freiburg, Freiburg, Germany; 6 Munich Cluster of Systems Neurology (SyNergy), Munich, Germany

## Dear Sirs,

It was an incidental observation in our tertiary outpatient dizziness center that patients with acquired bilateral vestibulopathy (BVP) rarely complain about being anxious about falling. This is surprising, since a bilateral vestibular deficit impairs postural stability and thus causes frequent falls [1], in particular during locomotion on uneven ground and in darkness when vision cannot substitute. In a controlled cross-sectional study, the rate of recurrent fallers was increased in BVP patients despite a low-to-normal fear of falling as determined by the Falls Efficiency Score International [2]. Two epidemiological studies had shown that patients with vestibular disorders with episodic vertigo, such as vestibular migraine and Menière’s disease, suffer from increased psychiatric comorbidity with anxiety and affective disorders, which was not the case in patients with BVP [3–5]. Furthermore, a recent study on the susceptibility to fear of heights in BVP patients did not demonstrate an increase in susceptibility (29% in BVP vs 28% of the general population) despite an objective higher risk of falling from height [6]. In contrast, patients with other vestibular syndromes showed an increased susceptibility to fear of heights.

This raised the question of whether intact vestibular function is relevant for distressing anxiety related to the particular type of vertigo or—in other words—whether loss of vestibular function reduces the liability to anxious behavior. This might be possible since Balaban and co-workers showed that there are neurological bases of links between balance control and anxiety. They described pathways that mediate autonomic control, vestibulo-autonomic interactions, and anxiety within a circuitry including a parabrachial nucleus network and its reciprocal connections with the central amygdaloid nucleus, the infralimbic and insular cortex, and the hypothalamus [7–10].

We carried out a survey on a total of 7083 outpatients with the key symptoms of vertigo, dizziness, and balance disorders all diagnosed in the German Center for Vertigo and Balance Disorders (DSGZ), Munich, Germany, in the years from 2010 to 2012 (group 1, *N* = 687) and 2015 to 2017 (group 2, *N* = 6396). All patients completed the Vertigo Handicap Questionnaire (VHQ [11]) to measure physical and psychosocial handicap due to vertigo and dizziness using 25 items. In addition to a sum-score, the VHQ allows two subscale scores, handicapped activity, and anxiety, to be generated. The latter was relevant for the current investigation. Moreover, all patients completed the Beck Anxiety Inventory (BAI; [12]) and the subscale Trait Anxiety of the State-Trait Anxiety Inventory (STAI; [13]) as further measures to assess anxiety in general. A total of 547 patients of group 1 were additionally examined with the Structured Clinical Interview for DSM-IV (SCID-I; [14]) to assess patients’ mental disorders and psychiatric comorbidity independently of their diagnoses given by the senior physician of the DSGZ (for details, see [15]). All subjects gave their written informed consent and signed a form confirming that they agree to further anonymous data analysis.

Linking diagnoses to VHQ forms was done with custom Java^®^9-based software combined with a MySQL^®^ Database by automatically analyzing medical documents. These documents were the result of outpatient routines at the DSGZ, including clinical neurological and neurootological examination, comprehensive neurophysiological diagnostics (including the video head impulse test, caloric testing, neuroorthoptic examination with cover test, adjustments of the subjective visual vertical, measurements of ocular torsion by fundus photographs, and posturography). All patients were finally seen by an experienced consultant supervisor from the center. Keywords for the common vestibular disorders, such as bilateral vestibulopathy (BVP), benign paroxysmal positional vertigo (BPPV), vestibular neuritis or unilateral vestibulopathy (UVP), vestibular paroxysmia (VP), Menière’s disease (MD), vestibular migraine (VM), and functional vertigo and dizziness (FD), were used for automatic filtering of the VHQ-linked forms. Statistical analysis was performed using R Core Team (2017), R-Statistics 3.4.3 [16]. Groups 1 and 2 were compared with a sensitivity analysis regarding demographic variables (age and gender) and vestibular diagnoses. The groups did not differ significantly in these variables (group 1: age 53.71 ± 15.78 years, female/male ratio 1.49; group 2: age 56.02 ± 24.41 years, female/male ratio 1.26).

**Fig. 1:**
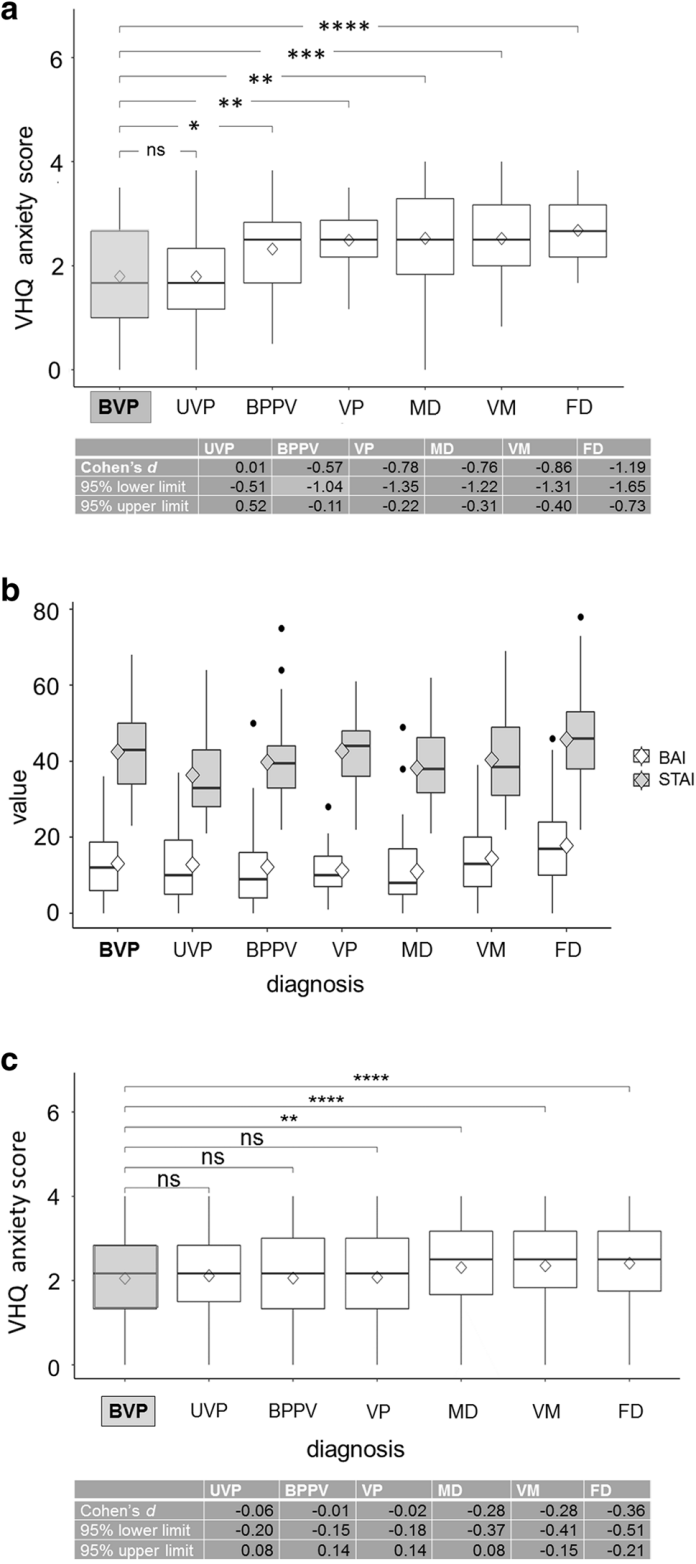
**a** Vertigo Handicap Questionnaire (VHQ) anxiety score boxplots from different vestibular syndromes of consolidated data sets from patient group 1 (*n* = 687) for each disease and a *t* test significance level compared to the reference group of bilateral vestibulopathy (BVP) (*p ≤ 0.05; **p ≤ 0.01, ***p ≤ 0.001; ****p ≤ 0.0001, ns *p* > 0.05). Vestibular syndromes were bilateral vestibulopathy (BVP), unilateral vestibulopathy/vestibular neuritis (UVP), benign paroxysmal positional vertigo (BPPV), vestibular paroxysmia (VP), Menière’s disease (MD), vestibular migraine (VM), and functional vertigo/dizziness (FD). Note that the scores were lowest for BVP and UVP and highest for MD, VM, and especially for FD. Median (horizontal solid line), mean (diamond square), boxplot rectangle (lower 25% quantile and higher 75% quantile). A quantification of the effect size magnitude was performed using the thresholds defined in Cohen [20], i.e., Cohen’s *d*. The magnitude was assessed using the thresholds provided in Cohen [20], i.e., |*d*| < 0.2 negligible, |*d*| < 0.5 small, |*d*| < 0.8 medium, otherwise large. Cohen’s *d* is given with 95% lower and upper limits for each disease. **b** For data set 1 (*n* = 687), in which the Structured Clinical Interview for DSM-IV (SCID-I) was performed, BAI (Beck Anxiety Inventory; white) and STAI (State-Trait Anxiety Inventory; grey) score value boxplots and outliers (black dots) are given for each disease to show that the low VHQ anxiety scores are not associated with psychiatric comorbidity (anxiety disorders). Cut-off ranges for the BAI have been suggested as follows: 0–7 (no or minimal anxiety), 8–15 (mild anxiety), 16–25 (moderate anxiety), and above 25 (severe anxiety). The STAI cut-off values proposed in the literature for clinically relevant anxiety effect are 39–40 for the original English version. **c** VHQ anxiety score boxplots from different vestibular syndromes of consolidated data sets from patient groups 1 and 2 (*n* = 3830) for each disease and a *t* test significance level compared to the reference group of BVP (**p ≤ 0.01; ****p ≤ 0.0001; ns *p* > 0.05). A quantification of the effect size magnitude was performed using the thresholds defined in Cohen [20], i.e., Cohen’s *d*. Cohen’s *d* is given with 95% lower and upper limits for each disease

This resulted in *n* = 575 patients with BVP, *n* = 672 with BPPV, *n* = 453 with UVP, *n* = 314 with VP, *n* = 514 with MD, *n* = 886 with VM, and *n* = 416 patients with FD for the total number of 3830 patients (groups 1 and 2) with consolidated data sets. The VHQ anxiety scores of the 687 patients of group 1 were lowest for patients with chronic BVP and UVP and highest for patients with FD (Fig. 1a). Significantly higher VHQ anxiety scores, as compared to BVP, were found for BPPV, VP, MD, VM, and FD; the disorders were listed according to the significance level. The low anxiety scores in BVP patients of group 1 were confirmed for the consolidated data sets of the pooled data of groups 1 and 2 (Fig. 1c). Regarding the scores on the BAI and the STAI-trait, patterns for the diagnostic groups showed no significant group differences (Fig. 1b), which means that the VHQ anxiety scores cannot simply be explained by psychiatric comorbidity of anxiety disorders. In terms of psychiatric diagnoses, 48.8% (*n* = 267) patients fulfilled a diagnosis according to SCID-I criteria. Of those, 28.9% (*n* = 158) were diagnosed with an anxiety disorder, 24.9% (*n* = 136) with a somatoform disorder, 19.0% (*n* = 104) with an affective disorder, 2.9% (*n* = 16) with a substance abuse disorder, and 0.7% (*n* = 4) with an eating disorder. These numbers were also reported by Lahmann and co-workers [5] who have analysed the prevalence of psychiatric disorders in the same sample (group 1).

The present data strongly support the view that a functioning peripheral vestibular system is the prerequisite for the development of anxiety related to vertigo and explains why anxiety scores were low in BVP patients. In contrast, all episodic vestibular syndromes as well as chronic functional dizziness revealed increased VHQ anxiety scores with the highest for MD, VM, and FD. One cannot simply compare our current data with the former epidemiological studies on psychiatric comorbidity in dizzy patients based on SCID-I diagnoses [5]. In the latter, a higher comorbidity incidence of anxiety/phobic, affective, and somatoform disorders was found especially in episodic vertigo syndromes but not in BVP. The low incidence of comorbidities in BVP patients was also seen in the current study.

It is well acknowledged in affective neuroscience that the vestibular and also the cerebellar systems are reciprocally connected to various anxiety and fear brain structures (for review: [17]). Such a network which probably involves the thalamus and hypothalamus provides the structure for coordination of sensorimotor behavior, emotional, higher vestibular cognitive, and visceral functions [17, 18]. Beside the basic circuitry connecting the emotional and vestibular systems (by a parabrachial nucleus network and its connections [7, 8]) an attempt has also been made at the cortical level—by use of transcranial direct current stimulation over the posterior parietal cortex—to reveal a link between anxiety and the vestibular system, i.e., the vestibulo-cortical dominance [19]. Thus, a close interaction between the vestibular and the emotional systems seems to exist at several lower as well as higher brain levels. However, our study does not allow interpretations as to specific structural and functional links between anxiety and the vestibular system. The major point we want to make here is the separation between psychiatric comorbidity in dizzy patients and anxiety triggered by particular vestibular disorders in patients who do not fulfill the diagnostic criteria of an associated psychiatric disorder. Nevertheless, in patients with a bilateral loss of peripheral vestibular function, both anxiety related to vertigo and psychiatric comorbidity are low.

## References

[1] Schniepp R, Schlick C, Schenkel F (2017). Clinical and neurophysiological risk factors for falls in patients with bilateral vestibulopathy. J Neurol.

[2] Schlick C, Schniepp R, Loidl V, Wühr M, Hesselbarth K, Jahn K (2016). Falls and fear of falling in vertigo and balance disorders: A controlled cross-sectional study. J Vest Res.

[3] Eckhardt-Henn A, Best C, Bense S (2008). Psychiatric comorbidity in different organic vertigo syndromes. J Neurol.

[4] Best C, Eckhardt-Henn A, Tschan R (2009). Psychiatric morbidity and comorbidity in different vestibular vertigo syndromes. Results from a prospective longitudinal study over one year. J Neurol.

[5] Lahmann C, Henningsen P, Brandt T (2015). Psychiatric comorbidity and psychosocial impairment among patients with vertigo and dizziness. J Neurol Neurosurg Psychiatry.

[6] Brandt T, Grill E, Strupp M, Huppert D (2018) Susceptibility to fear of heights in bilateral vestibulopathy and other disorders of vertigo and balance. Front Neurol. 10.3389/fneur.2018.00406 10.3389/fneur.2018.00406 PMC5997824 29928252

[7] Balaban CD, Thayer JF (2001). Neurological bases for balance-anxiety links. Anxiety Disord.

[8] Balaban CD, Jacob RG, Furman JM (2011). Neurologic bases for comorbidity of balance disorders, anxiety disorders, and migraine: Neurotherapeutic implications. Exp Rev Neurother.

[9] Staab J, Balaban CD, Furman JM (2013). Threat assessment and locomotion: clinical applications of an integrated model of anxiety and postural control. Seminars Neurol.

[10] Coelho CM, Balaban CD (2015). Visuo-vestibular contributions to anxiety and fear. Neurosci Biobehav Rev.

[11] Yardley L, Masson E, Verschuur C, Haacke N, Luxon L (1992). Symptoms, anxiety and handicap in dizzy patients: development of the vertigo symptom scale. J Psychosom Res.

[12] Steer RA, Rissmiller DJ, Ranieri WF, Beck AT (1993). Structure of the computer-assisted Beck Anxiety Inventory with psychiatric inpatients. J Pers Assess.

[13] Laux L, Glanzmann P, Schaffner P, Spielberger CD (1981). Das state-trait-Angstinventar (STAI) [The state-trait anxiety inventory].

[14] Wittchen H, Wunderlich U, Gruschwitz S, Zaudig M (1997). SCID I: structured clinical interview for DSM-IV: Axis I: mental disorders.

[15] Limburg K, Dinkel A, Schmid-Mühlbauer G (2018). Neurologists‘ assessment of mental comorbidity in patients with vertigo and dizziness in routine clinical care—comparison with a structured clinical interview. Front Neurol.

[16] R Core Team (2017) R: A language and environment for statistical computing. R Foundation for Statistical Computing, Vienna, Austria. URL https://www.R-project.org/

[17] Hilber P, Cendelin J, Le Gall A (2019). Cooperation of the vestibular and cerebellar networks in anxiety disorders and depression. Prog Neuropsychopharmacol Biol Psychiatry.

[18] Brandt T, Dieterich M (2019). Thalamocortical network: a core structure for integrative multimodal vestibular functions. Curr Opin Neurol.

[19] Bednarczuk NF, Casanovas Ortega M, Fluri AS, Arshad Q (2018). Vestibulo-cortical hemispheric dominance: the link between anxiety and the vestibular system?. Eur J Neurosci.

[20] Cohen J (1988). Statistical power analysis for the behavioral sciences.

